# Investigation of electropolishing characteristics of tungsten in eco-friendly sodium hydroxide aqueous solution

**DOI:** 10.1007/s40436-020-00309-y

**Published:** 2020-05-26

**Authors:** Wei Han, Feng-Zhou Fang

**Affiliations:** 1 Centre of Micro/Nano manufacturing Technology (MNMT-Dublin), University College Dublin, Dublin 4, Ireland; 2 State Key Laboratory of Precision Measuring Technology and Instruments, Centre of Micro/Nano Manufacturing Technology (MNMT), Tianjin University, Tianjin, 300072 People’s Republic of China

**Keywords:** Electropolishing, NaOH solution, Surface roughness, Tungsten, Etching

## Abstract

In this study, an eco-friendly electrolyte for electropolishing tungsten and the minimum material removal depth on the electropolished tungsten surface are investigated using an electrochemical etching method. Using a concentrated acid electrolyte, the polarization curve and current density transient are observed. For a NaOH electrolyte, the effects of interelectrode gap and electrolyte concentration on electropolishing are investigated. The differences in electropolishing characteristics are compared among different electrolyte types. Microholes are etched on the electropolished tungsten surface to determine the minimum material removal depth on the tungsten surface. Experimental results indicate the color effect due to a change in the thickness of the oxide film on the tungsten surface after electropolishing with a concentrated acid electrolyte. The surface roughness decreases with the interelectrode gap width owing to the increased current density when using the NaOH electrolyte. However, the electropolishing effect is less prominent with a significantly smaller gap because the generated bubbles are unable to escape from the narrow working gap in time. A material removal depth of less than 10 nm is achieved on the tungsten surface in an area of diameter 300 µm, using the electrochemical etching method.

## Introduction

Tungsten has the highest melting point of all available metals with a melting temperature at 3 422 °C and is applied extensively in various fields, such as arc-welding electrodes [1, 2] and heat-resistant coatings [3]. Furthermore, it is the most typically used material for preparing scanning probe microscopy and scanning tunneling spectroscopy probes owing to its good physical and chemical properties [4, 5]. Moreover, tungsten has high stiffness and good electrical conductivity and is generally used as tool electrodes in electrical discharge machining [6] and electrochemical machining (ECM) [7, 8].

As a hard-brittle material, tungsten is difficult to machine using conventional machining methods, such as cutting and grinding, owing to its low machinability [9, 10]. It has been reported that the ultraprecision diamond cutting of tungsten is significantly affected by the adhesion of tungsten to the tool, rapid tool wear, and brittle fracture [11]. Hence, tungsten is typically machined using nonconventional machining methods. Wire electrical discharge machining (WEDM) is an effective solution for machining hard materials such as zirconium, titanium, and tungsten carbide, which are difficult to machine using conventional machining methods [12]; additionally, it is an alternative for tungsten machining [13, 14]. However, WEDM is a thermal process in which the material is removed by melting, and vaporization and the formation of a heat-affected layer on the machined surface are inevitable [15]. In addition, tool wear due to thermal processes occurs in WEDM, resulting in a deterioration in machining precision.

To polish tungsten, chemical-mechanical polishing (CMP) is often used, in which synergetic effects of chemical and mechanical interactions are involved to achieve global planarization [16, 17]. Bielmann et al. [18] reported that the tungsten removal rate increased with decreasing particle size and increasing solid loading. Larsen-Basse and Liang [19] studied the contributions of abrasion in the CMP of tungsten and concluded that it was a synergistic process of passive film removal by abrasives and the reformation of a film by the action-passive reaction of a bare surface with a slurry. Although a chemical reaction is involved in CMP, its fundamental is based on the traditional mechanical polishing process [20]. Slurry particles and polishing byproducts that are pressed onto the workpiece surface owing to mechanical forces are serious defects. It is still challenging to directly apply a polished workpiece by CMP because of the dirty surface; therefore, a post-CMP cleaning process is required. In addition, the low material removal rate and significant slurry consumption render CMP a high-cost polishing method [21].

Moreover, electropolishing, also known as electrochemical polishing, anodic polishing, or electrolytic polishing, is a promising method for polishing tungsten because the material is removed by electrochemical reactions, which is a non-mechanical contact and damage-free processes without considering the hardness and brittleness of a workpiece [22–24]. The electropolishing method has been applied extensively in the surface treatment of metals with complex features, such as coronary stents and niobium superconducting radio frequency cavities. As CMP, which is widely used for polishing tungsten, is a high-cost finishing method owing to its requirement of a large amount of consumed slurry, the electropolishing of tungsten in NaOH electrolyte is an effective alternative as electropolishing is an easy and simple approach. High polishing efficiency can be achieved by increasing the current density and no post-treatments are required compared with CMP. Schubert et al. [25] studied the anodic dissolution behavior of tungsten carbide in an alkaline electrolyte under electrochemical machining conditions and discovered that near the interface, an adherent, supersaturated, viscous film of polytungstates was formed, which was then continuously dissolved and reproduced. The anodic dissolution process of electropolishing tungsten has been studied by Wang et al. [21], and they discovered that electropolishing tungsten in a NaOH aqueous solution with different applied potentials could be categorized into three stages: etching, brightening, and pitting. However, it is noteworthy that electropolishing tungsten in a NaOH aqueous solution differs from the conventional electropolishing conducted in a concentrated acid electrolyte owing to the different physical and chemical characteristics of electrolytes used. In a concentrated acid electrolyte, a thick viscous film layer is formed on the workpiece surface because the dissolved metal ions cannot diffuse into the viscous bulk electrolyte in time [26]. Previous studies have not focused on the differences between electropolishing tungsten in a NaOH aqueous solution and the conventional electropolishing in a concentrated acid electrolyte [21, 27].

In this study, the electropolishing characteristics of tungsten were investigated using different types of electrolytes, i.e., the conventional concentrated acid electrolyte and a NaOH aqueous solution. Subsequently, the minimum value of the material removal depth on the tungsten surface was determined based on the electropolished tungsten surface using an electrochemical etching method. For the study using the concentrated acid electrolyte, the polarization curve and current density transient during electropolishing tungsten were characterized. For the study using the NaOH aqueous solution, the effects of the interelectrode gap width and electrolyte concentration on the electropolishing of tungsten were investigated. Holstein et al. [28] reported the achievable minimum dimension of 100 µm of metallic tungsten adhesion elements in round and square-edged variations using the ECM method. In this study, microholes of diameter 300 µm were etched on an electropolished tungsten surface to investigate the minimum material removal depth on the tungsten surface using the ECM method.

## Experimental approach

### Material and solution

Table 1 shows the electrolytes and electrodes used for electropolishing tungsten. The concentrated acid electrolyte was composed of a phosphoric acid aqueous solution (H_3_PO_4_, 81% in volume ratio) and glycerol in the volume ratio of 3:1. The NaOH electrolyte was prepared by dissolving NaOH powder in distilled water to the concentrations of 0.27 mol/L and 0.5 mol/L. The same workpiece and tool electrode were used for the two types of electrolytes. The workpiece electrode was tungsten wire with a diameter of 1 mm and was mounted in a resin of diameter 30 mm to expose only the end surface. Hence, the effective surface area was a circle of diameter 1 mm in the electropolishing. The tool electrode was a copper sheet measuring 30 mm × 10 mm × 0.1 mm, and one side was insulated by tape to reduce the effects of stray currents.

**Table 1:** Electrolytes and electrodes used for electropolishing tungsten

Electrolyte type	Composition	Workpiece electrode	Tool electrode
Concentrated acid electrolyte	H_3_PO_4_ (81% v:v) : Glycerol = 3:1	Tungsten wire (*φ*1 mm)	Copper sheet (30 mm × 10 mm × 0.1 mm)
NaOH electrolyte	NaOH aqueous solution (0.27 mol/L, 0.5 mol/L)	Tungsten wire (*φ*1 mm)	Copper sheet (30 mm × 10 mm × 0.1 mm)

Table 2 shows the electrolyte and electrodes used for the nanoscale etching of tungsten. The electropolished tungsten was used as a workpiece with the exposed end surface measuring 1 mm in diameter. Copper wire of diameter 300 µm was used as the tool electrode and was mounted in a resin of diameter 30 mm. The resin was visible owing to the easy observation of the electrodes’ relative position. The mounted copper wire was ground and mechanically polished in sequence to obtain a smoother surface. The electropolishing of copper was performed in H_3_PO_4_ aqueous solution with a concentration of 81%, and the tool electrode was the same as that shown in Table 1.

**Table 2:** Electrolyte and electrodes used for the nanoscale etching of tungsten

Electrolyte	Workpiece electrode	Tool electrode
NaOH aqueous solution (0.5 mol/L)	Electropolished tungsten wire (*φ*1 mm)	Copper wire (*φ* 300 µm)

The surface morphology was characterized using a digital microscope (VHX-5000), and the surface topography was measured by a noncontact optical profile (NPFLEX). Contact mode atomic force microscopy (MFP-3D) was used for the analysis of the image topography.

### Experimental method

**Fig. 1:**
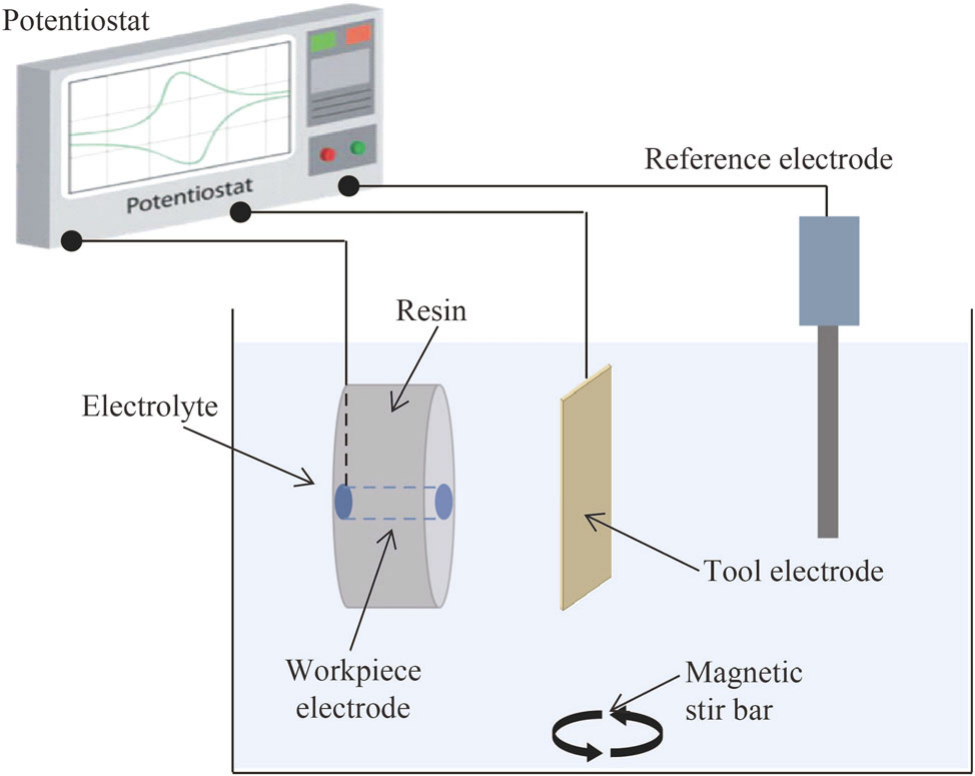
Experimental setup used for electropolishing

Figure 1 shows the experimental setup used for electropolishing in this study. A typical electrochemical cell with three electrodes was used, and the reference electrode was a Ag/AgCl reference element surrounded by an electrolyte of 4 mol/L KCl aqueous solution saturated with AgCl. A potentiostat/galvanostat (CS310) was used to supply the applied potential between the electrodes. A magnetic stir bar was used to stir the electrolyte at a stirring speed of 667 r/min. The setup show in Fig. 1 was also used for the nanoscale etching of tungsten surface with a copper tool electrode mounted in resin.

**Fig. 2:**
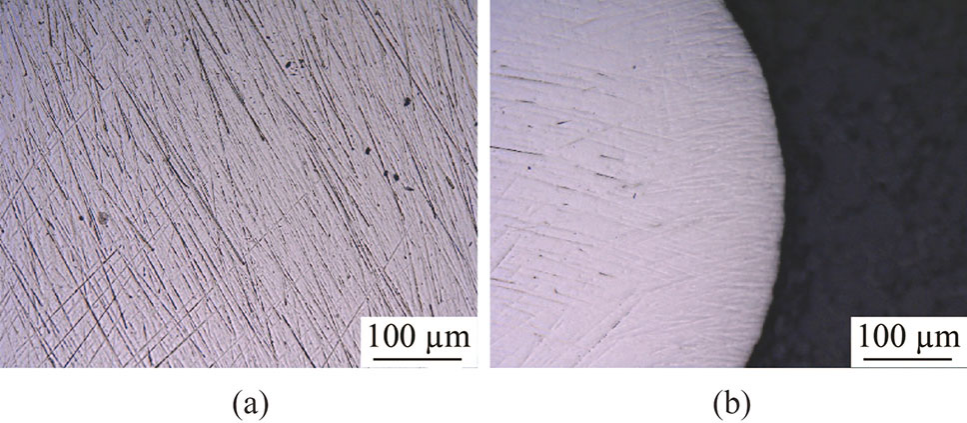
**a** Ground and **b** mechanical polished tungsten surface

It is crucial to reduce the initial surface roughness of a workpiece before electropolishing because the electropolishing method is limited in terms of polishing ability to improve the surface roughness [24]. The final electropolished surface is better with a lower initial surface roughness [29]. Therefore, the workpiece electrodes used in electropolishing were ground and mechanically polished prior to electropolishing to obtain a better electropolishing effect. They were uniformly ground with 600, 1 200, and 2 500 grit abrasive sandpaper sequentially and then mechanically polished using a colloidal silica polishing suspension of micro sizes 3 µm and < 1 µm. Figure 2 shows the ground and mechanically polished tungsten surfaces. Because tungsten is a significantly hard material, scratches that form due to grinding could not be removed completely by the subsequent mechanical polishing, as shown in Fig. 2b. It was observed that the number of scratches in the vicinity of the edge was less than that at the center because the refresh of colloidal silica polishing suspension was more efficient during mechanical polishing.

Table 3 shows the experimental conditions used to study the effects of the interelectrode gap width and electrolyte concentration on the electropolishing of tungsten in NaOH electrolyte. The interelectrode gap width was varied to 0.15, 0.5, 1.0, and 1.5 mm, and the concentrations of the NaOH aqueous solution were 0.27 mol/L and 0.5 mol/L. To determine the optimum applied potential, the potential was reduced step by step from the maximum value of 10 V supplied by the potentiostat/galvanostat while monitoring for a significant oscillation in the current density; a final potential of 8 V was decided.

**Table 3:** Experimental conditions used to study the influences of interelectrode gap width and electrolyte concentration on the electropolishing effect of tungsten in NaOH electrolyte

Parameter	Value
Applied potential/V	8
Electrolyte (NaOH aq.) concentration/ (mol·L^−1^)	0.27, 0.5
Electropolishing duration/s	200, 300, 400, 500
Interelectrode gap widths/mm	0.15, 0.5, 1.0, 1.5

Table 4 shows the experimental conditions used for the nanoscale etching of the tungsten surface. The electrolyte was a NaOH aqueous solution of concentration 0.5 mol/L. The interelectrode gap width was 0.3 mm, and the stirring speed of the magnetic stir bar was 667 r/min. The etching durations were 35 s and 75 s. The surface quality of the tool electrode is critical in nanoscale etching because the surface shape of the tool electrode can be converted into the machined surface [30]. The copper tool electrode, which was used as a tool electrode for the nanoscale etching of the electropolished tungsten, was ground and mechanically polished sequentially based on the methods for preparing tungsten. Subsequently, electropolishing was performed to reduce the grain boundaries on the copper surface by optimizing the process parameters.

**Table 4:** Experimental conditions used for the nanoscale etching of tungsten

Parameter	Value
Applied potential/V	5
Electrolyte (NaOH aq.) concentration/ (mol·L^−1^)	0.5
Inter-electrode gap width /mm	0.3
Stir speed/(r·min^−1^)	667
Etching duration/s	35, 75

**Fig. 3:**
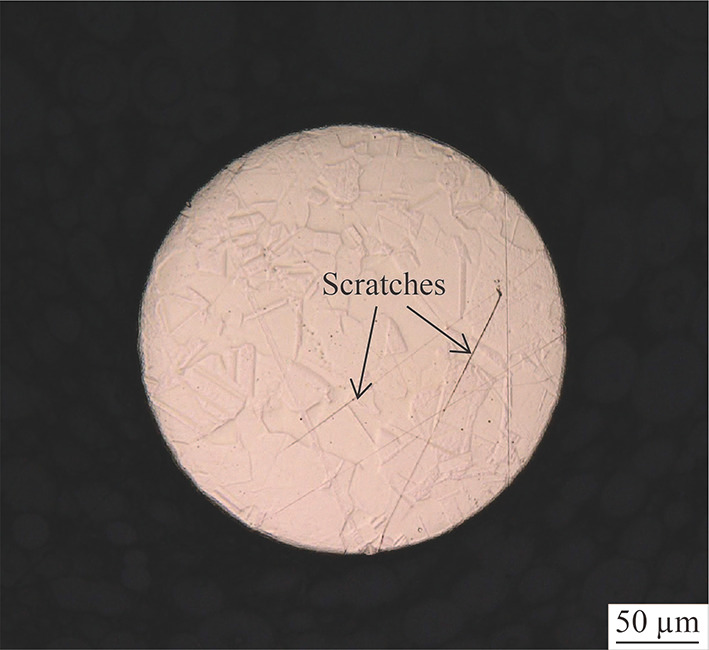
Copper tool surface after mechanical polishing

Figure 3 shows the copper tool surface after grinding and mechanical polishing. Grain boundaries can be observed clearly on the surface. Moreover, scratches appeared after the mechanical polishing. They were typically generated on the copper surface by the grinding process and most of them disappeared after the mechanical polishing. The scratches shown in Fig. 3 might have generated during grinding, and the mechanical polishing failed to remove some significantly deep scratches caused by large abrasives during grinding. Furthermore, this might be caused by contaminative particles, such as abrasives from grinding, during the mechanical polishing. Because copper has a low hardness, scratches can be formed easily even when only a few contaminative particles exist in the polishing suspension. The mechanically polished surface shown in Fig. 3 was treated by electropolishing to obtain a significantly smooth surface.

## Results and discussion

### Electropolishing tungsten in a concentrated acid electrolyte

**Fig. 4:**
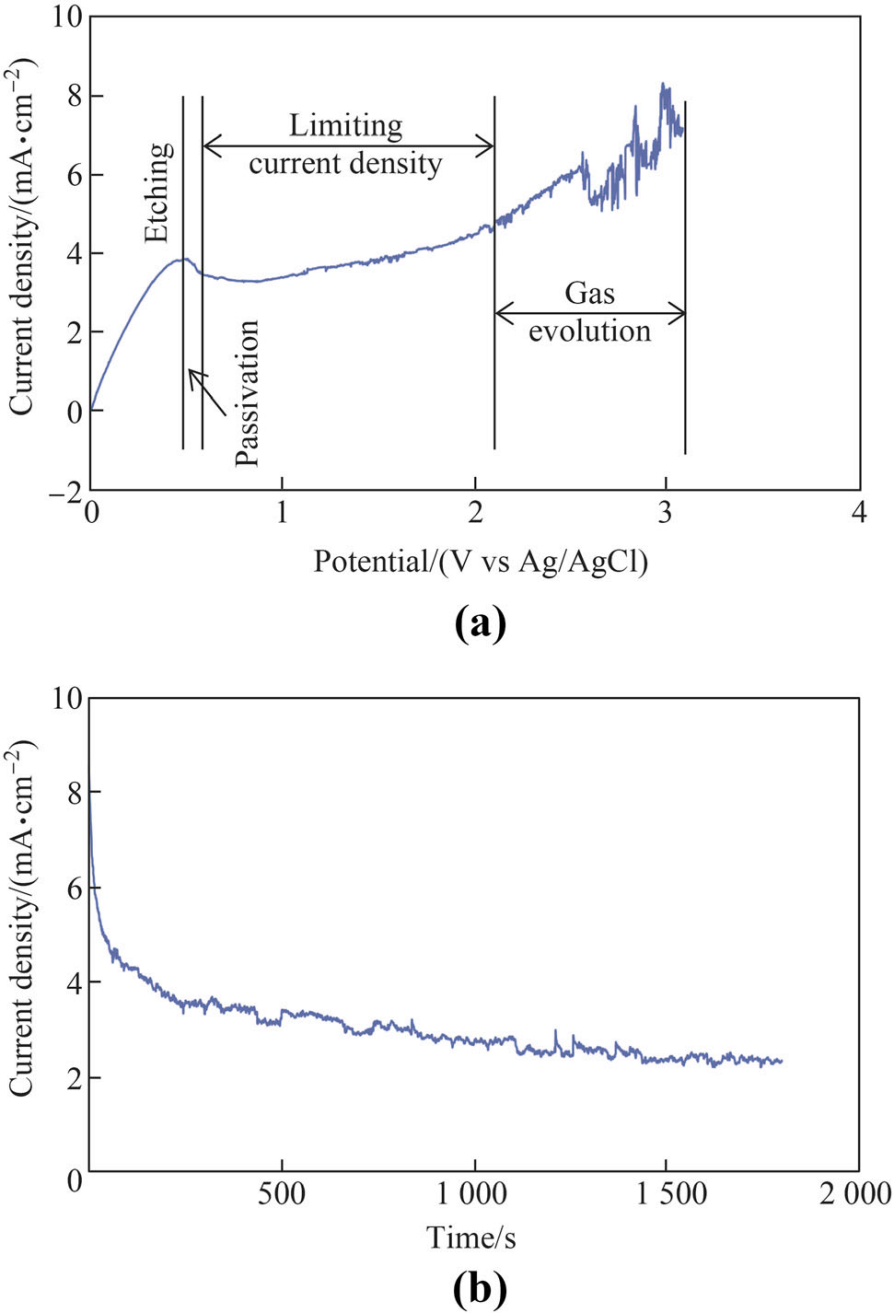
**a** Polarization curve measured with the scan rate of − 20 mV/s and potential range of 3–0 V and **b** current density transient with the applied potential of 2 V and electropolishing duration of 30 min

Figure 4 shows the polarization curve that was obtained by sweeping the applied potential from 3 V to 0 V (vs Ag/AgCl_sat_) at a scan rate of − 20 mV/s, and the current density transient measured in the concentrated acid electrolyte. It has been reported that the potential must be swept from the positive values in the cathodic direction to obtain reproducible measurements [31]. Four typical potential regions, i.e., etching, passivation, limiting current density plateau, and gas evolution were observed on the polarization curve, as shown in Fig. 4a. The current density oscillated with a large amplitude in the gas evolution region because of the evolution of oxygen gas from the tungsten surface. Because the best electropolishing effect is typically obtained in the limiting current density plateau region [24], 2 V was applied as the potential for electropolishing. Figure 4b shows the typical current density transient in electropolishing. The decrease in the current density at the initial stage was due to the generation of a thick oxide film layer on the workpiece surface. Subsequently, it stabilized with increasing electropolishing duration because of the balance achieved between the generation and dissolution of the oxide film layer.

**Fig. 5:**
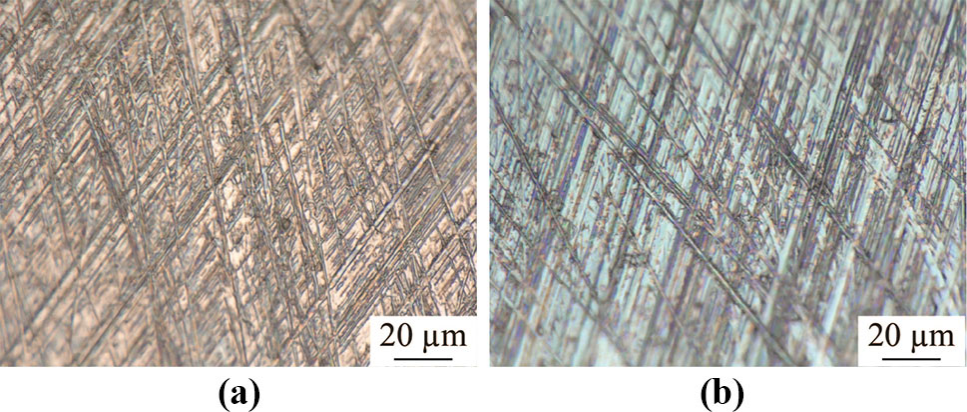
Images of tungsten surface **a** before and **b** after electropolishing

Figure 5 shows the images of the tungsten surface before and after electropolishing. The surface finish showed almost no improvement; therefore, it was concluded that a slight electropolishing effect occurred in tungsten electropolishing when the conventional concentrated acid electrolyte composed of 81% concentration H_3_PO_4_ solution and glycerol in the volume ratio of 3:1. Because the thick oxide film caused a high interelectrode gap resistance, the current density was significantly low, as shown in Fig. 4, resulting in a low material removal rate and the electropolishing effect. The overall electrode reactions can be expressed by the following chemical reactions when electropolishing tungsten using the concentrated acid electrolyte [32, 33]

1 $${\text{Cathode}}\quad 2{\text{H}}^{ + } + 2{\text{e}}^{ - } \to {\text{H}}_{2} \left( {\text{g}} \right)$$

2 $${\text{Anode}}\quad {\text{W}}\left( {\text{s}} \right) + 3{\text{H}}_{2} {\text{O}} = {\text{WO}}_{3} \left( {\text{s}} \right) + 6{\text{H}}^{ + } + 6{\text{e}}^{ - }$$

3 $${\text{WO}}_{3} \left( {\text{s}} \right) + {\text{H}}_{2} {\text{O}} \to {\text{WO}}_{3} \cdot{\text{H}}_{2} {\text{O}}\left( {\text{s}} \right) = {\text{H}}_{2} {\text{WO}}_{4} \left( {\text{aq}} \right)$$

The dissolution of the oxide film layer by reaction (3) is an extremely slow process, thereby resulting in the low material removal rate in electropolishing. It has been discovered that WO_3_ can be dissolved using H^+^ when the solution pH is lower than 1, based on the following reaction [33, 34]

4 $${\text{Cathode}}\quad {\text{WO}}_{3} \left( {\text{s}} \right) + {\text{H}}^{ + } \to {\text{WO}}_{2} {\text{OH}}^{ + } \left( {\text{aq}} \right)$$

However, the pH of the 81% H_3_PO_4_ aqueous solution was between 1.0–1.6, resulting in the dissolution of the oxide film layer in a H_2_O-assisted process, as shown in reaction (3), with an extremely low dissolution rate; this is verified by the electropolished results shown in Fig. 5. Meanwhile, tungsten can be electropolished in a concentrated acid electrolyte of pH lower than 1 owing to the dissolution of the oxide film layer by reaction (4), which necessitates more investigation in the future.

Moreover, Fig. 5 shows that the surface color of tungsten changed after electropolishing though the electropolishing effect was not evident. Evans et al. [35] studied the composition and thickness of the colored oxide films on a stainless steel surface after they were immersed in a solution containing chromic and sulfuric acids at 70 °C. It was reported that the colors on the steel surface area were produced by interference between the light reflected from the metal/film interface and that reflected from the film/air interface. The film thickness is changed as the reaction proceeds in the solution, thus producing changes in color. The electropolishing process failed to improve the tungsten surface significantly; however, the thickness of oxide film was changed, resulting in the different colors shown in Fig. 5b. The change of the oxide film layer could also be verified by the initial decrease in the current density transient shown in Fig. 4b.

### Electropolishing tungsten in NaOH aqueous solution

**Fig. 6:**
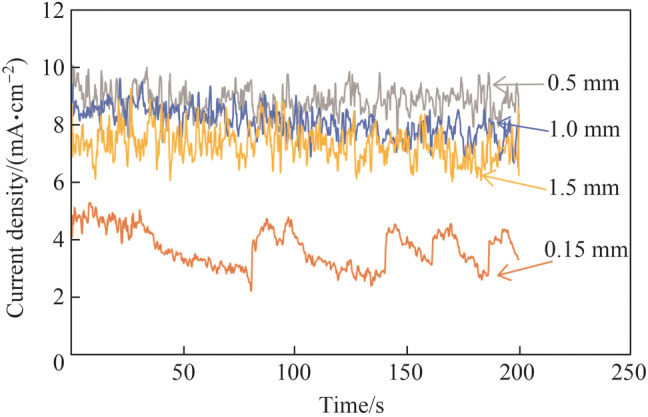
Current density transitions with different interelectrode gap widths

Figure 6 shows the current density transitions with different interelectrode gap widths. At the large gap width of 1.5 mm, the current density decreased owing to the high gap resistance. It was discovered that the current density decreased significantly at the gap width of 0.15 mm. This occurred because the gas produced by electrochemical reactions could not dissipate from the narrow working gap efficiently, resulting in a higher gap resistance and lower current density. Moreover, four spikes in the current density transition were observed at 82, 140, 161, and 187 s at the gap width of 0.15 mm. It was speculated that as the total number of generated gas bubbles increased with the electropolishing duration, some small gas bubbles merged to form a bigger bubble and then escaped from the narrow working gap under the action of electrolyte stirring, resulting in some fresh electrolytes flowing into the narrow working gap. Hence, the current density increased quickly owing to the decreased working gap resistance. Subsequently, the current density decreased slowly after the spike, as shown in Fig. 6, because new gas bubbles were generated and gathered in the narrow working gap again. Shimasaki and Kunieda [36] observed the gap phenomenon of ECM using transparent electrodes made of SiC single crystal as the cathode and discovered that the working gap was filled with gas bubbles within several milliseconds, which significantly affected the gap resistance and current density. Hence, for the electropolishing of tungsten, the interelectrode gap width should be optimized to avoid the effects of gas bubbles in a narrow working gap.

Moreover, it was discovered that the current density transitions in the NaOH aqueous solution differed from those in the concentrated acid electrolyte shown in Fig. 4b. Firstly, the current density was much higher when the NaOH aqueous solution was used. This might be due to the higher applied potential or the dissolution of the oxide film layer in the NaOH aqueous solution. When electropolishing tungsten with a NaOH aqueous solution, the overall electrode reactions can be expressed as follows [21, 37]

5 $${\text{Cathode}}\quad 6{\text{H}}_{2} {\text{O}} + 6{\text{e}}^{ - } \to 3{\text{H}}_{2} \left( {\text{g}} \right) + 6{\text{OH}}^{ - }$$

6 $${\text{Anode}}\quad {\text{W}}\left( {\text{s}} \right) + 6{\text{OH}}^{ - } \to {\text{WO}}_{3} \left( {\text{s}} \right) + 3{\text{H}}_{2} {\text{O}} + 6{\text{e}}^{ - }$$

7 $${\text{WO}}_{3} \left( {\text{s}} \right) + 2{\text{OH}}^{ - } \to {\text{WO}}_{4}^{2 - } + {\text{H}}_{2} {\text{O}}$$

The oxide film layer can be dissolved rapidly on the tungsten surface by reaction (7) in the NaOH aqueous solution; subsequently, an obvious electropolishing effect can be obtained. An air-formed oxide film layer is generated on the tungsten surface before the electropolishing; therefore, reactions (3) and (7) should occur first when electropolishing tungsten in a concentrated acid electrolyte and NaOH aqueous solution, respectively. However, the oxide film layer can be easily dissolved by reaction (7) and the dissolution rate is extremely low owing to reaction (3). Therefore, the electropolishing of tungsten indicated a higher current density in the NaOH aqueous solution, as shown in Figs. 4 and 6.

**Fig. 7:**
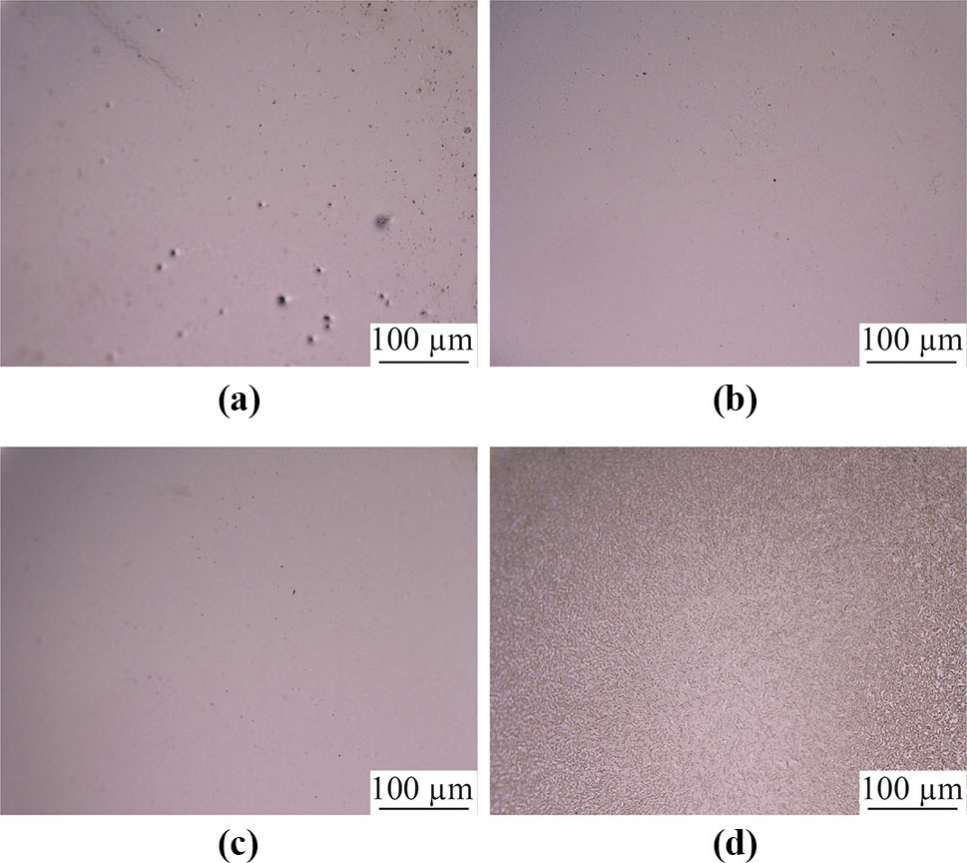
Electropolished tungsten surfaces with different interelectrode gap widths of **a** 0.15 mm, **b** 0.5 mm, **c** 1.0 mm and **d** 1.5 mm

**Fig. 8:**
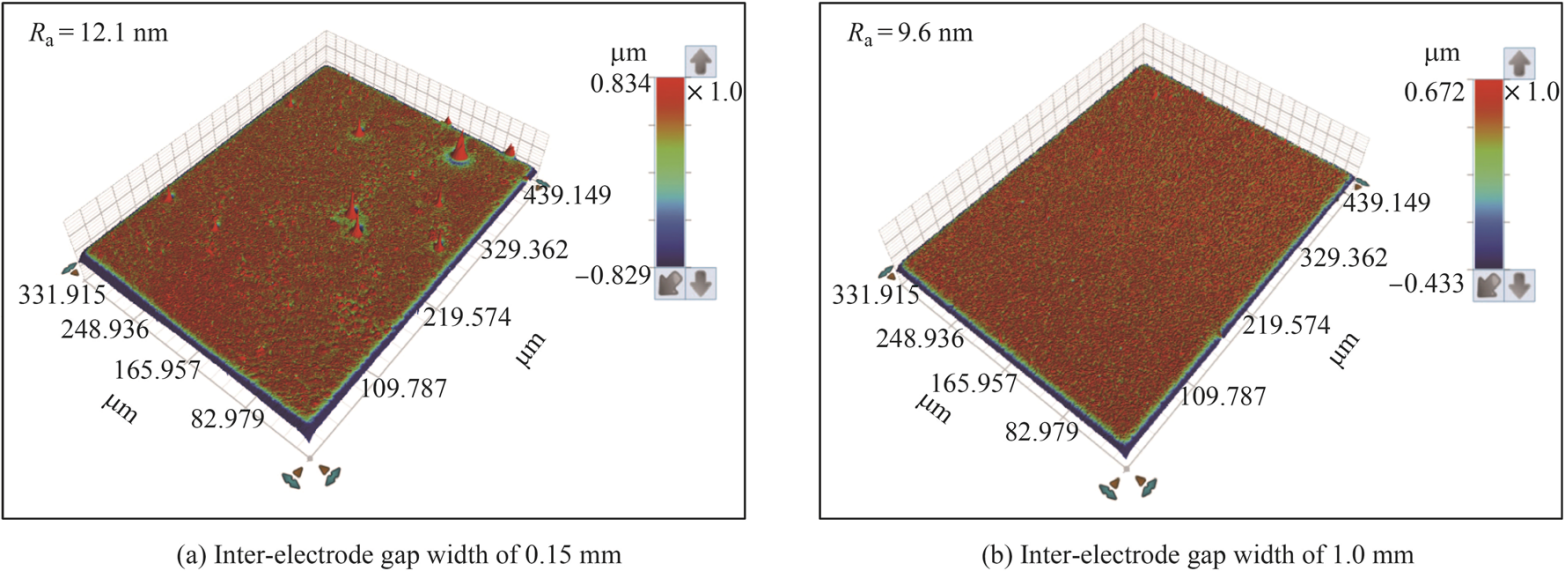
Topographies of electropolished tungsten surfaces with different interelectrode gap widths

**Fig. 9:**
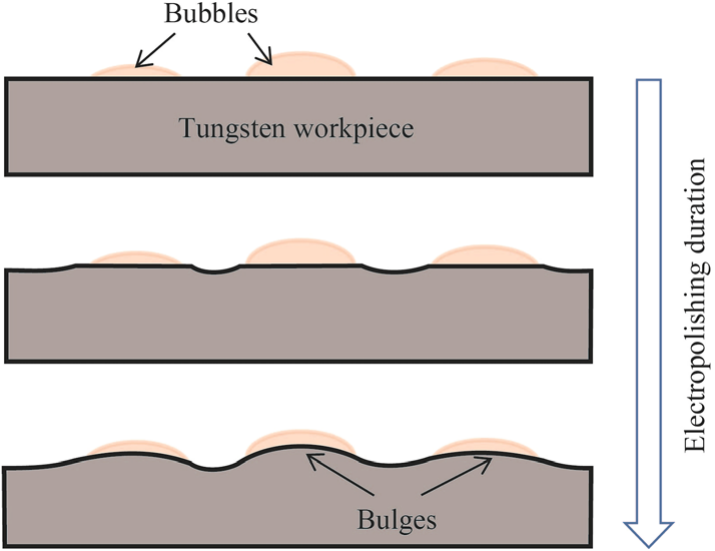
Protection effect of gas bubbles on the tungsten surface in electropolishing

**Fig. 10:**
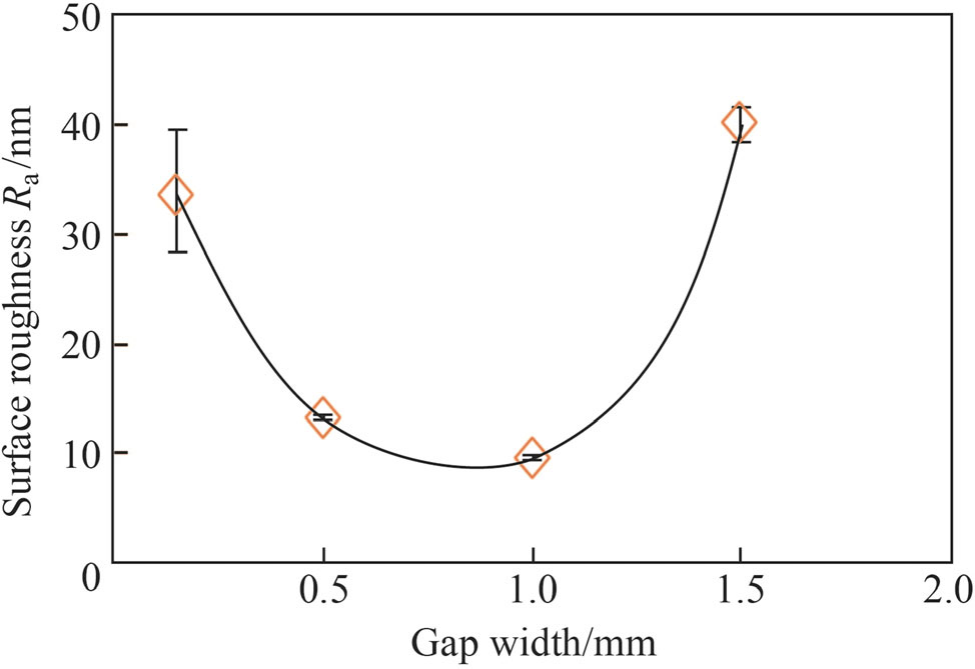
Surface roughness *R*_a_ obtained with different interelectrode gap widths

Figure 7 shows the electropolished tungsten surfaces with different interelectrode gap widths. Figure 8 shows the topographies of the electropolished tungsten surfaces with the interelectrode gap widths of 0.15 mm and 1.0 mm. With the interelectrode gap width of 0.15 mm, some bulges were observed, as shown in Figs. 7a and 8a. The gas bubbles attached to these positions and protected the surface against dissolution during electropolishing process. Figure 9 shows the schematic diagram of the protection effect. In addition, Fig. 7d shows that the electropolished surface was rougher at the interelectrode gap width was 1.5 mm because of the lower current density—a higher current density generates a better surface finish in ECM. With the optimized interelectrode gap width of 1.0 mm, the surface roughness *R*_a_ decreased to 9.6 nm, as shown in Fig. 8b. Figure 10 shows the *R*_a_ obtained using different interelectrode gap widths, and the optimized interelectrode gap of 1.0 mm generated the lowest surface roughness *R*_a_.

**Fig. 11:**
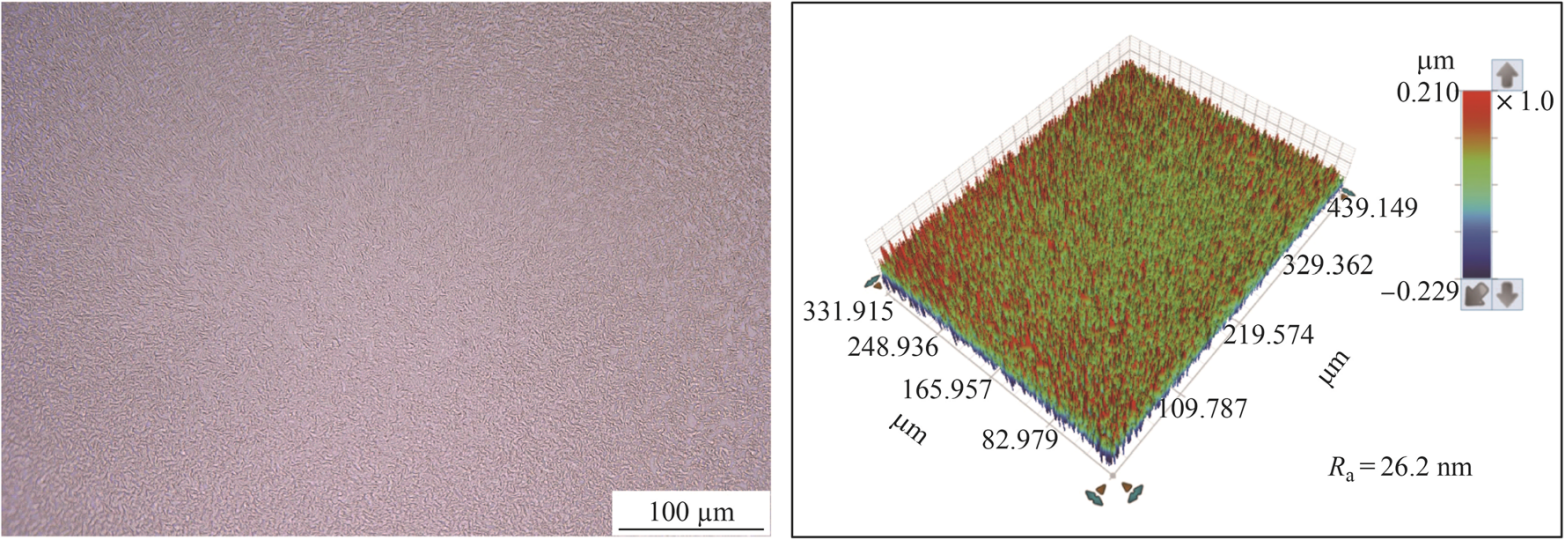
Electropolished tungsten surface with the NaOH concentration of 0.27 mol/L and duration of 200 s

**Fig. 12:**
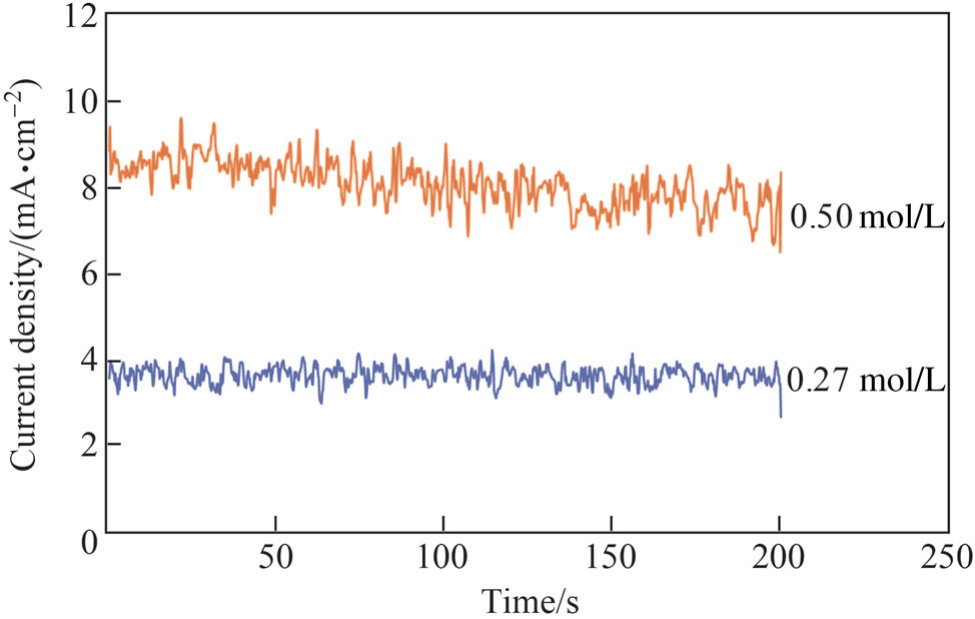
Current density transitions with the NaOH concentrations of 0.27 mol/L and 0.5 mol/L

**Fig. 13:**
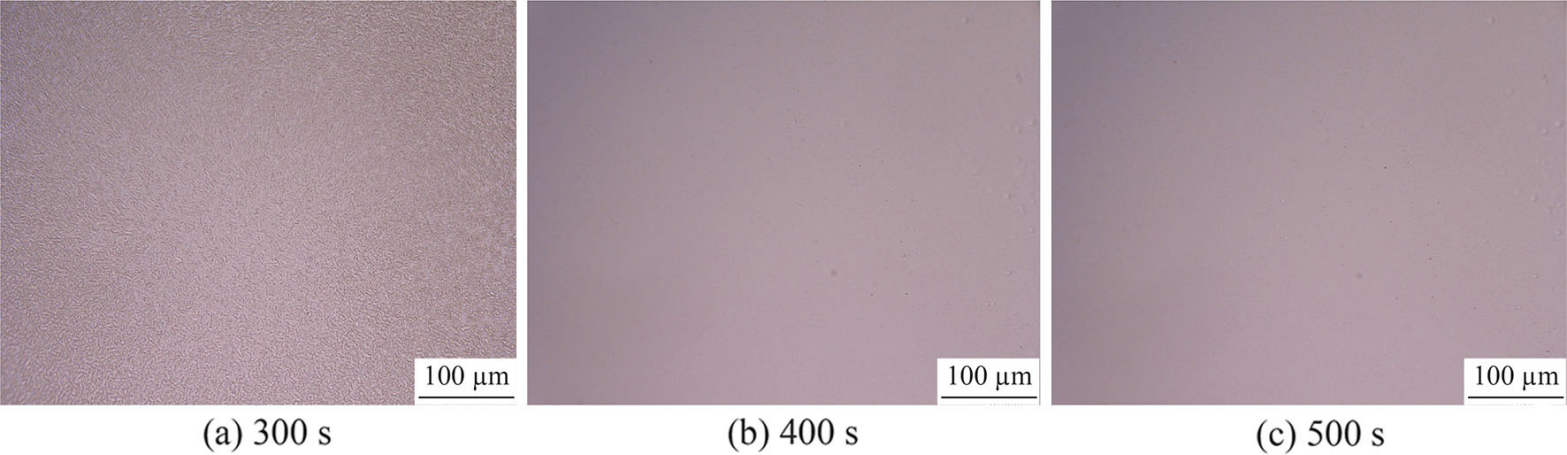
Electropolished tungsten surfaces with different electropolishing durations and the NaOH electrolyte concentration of 0.27 mol/L

**Fig. 14:**
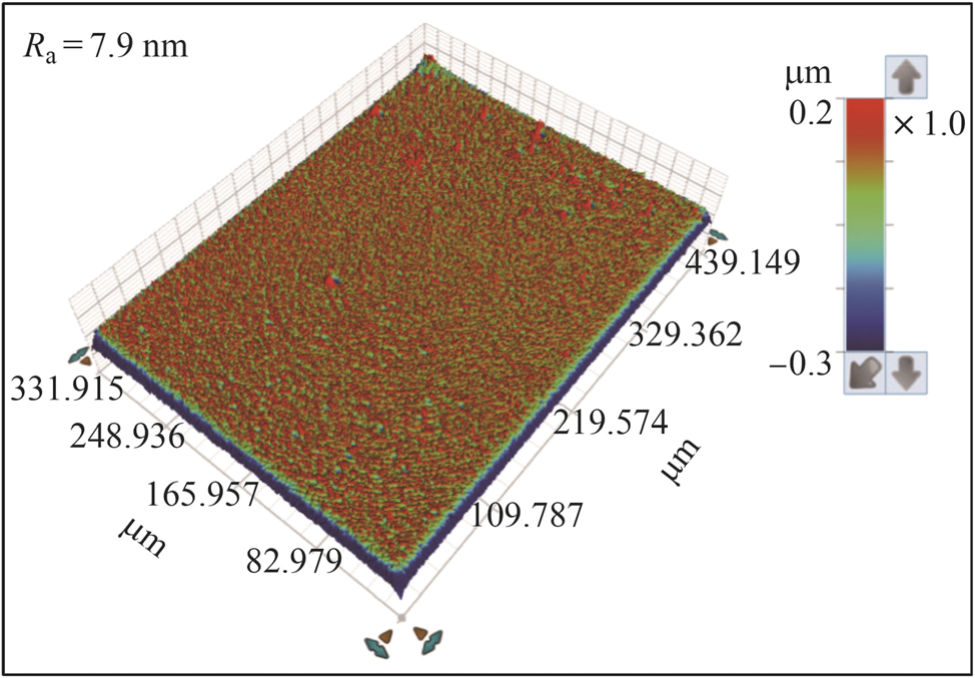
3D Plot of electropolished tungsten surface with a duration of 400 s and NaOH concentration of 0.27 mol/L

**Fig. 15:**
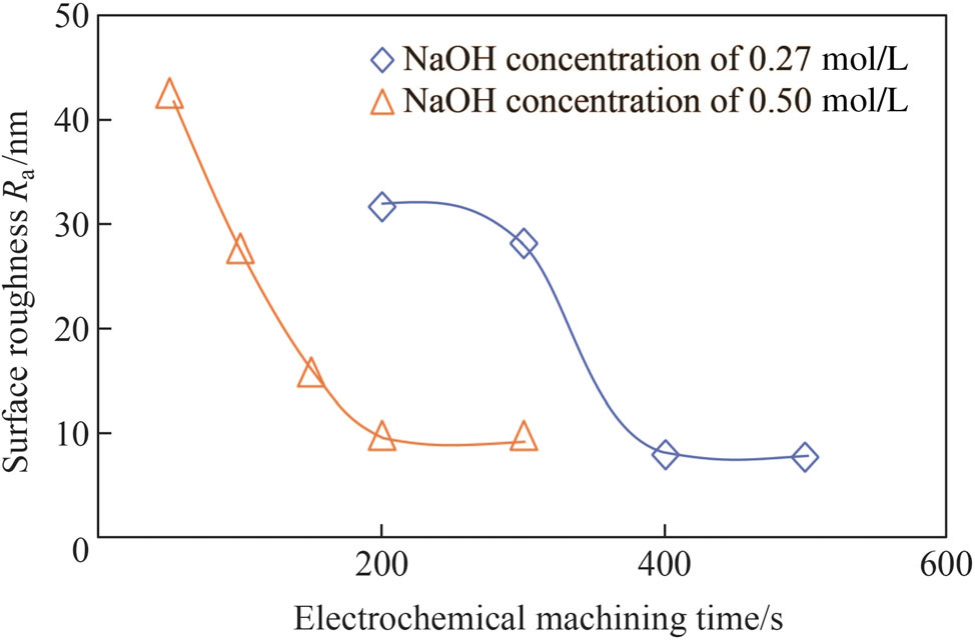
Surface roughness *R*_a_ obtained with different NaOH concentrations

Figure 11 shows the electropolished tungsten surface with the NaOH concentration of 0.27 mol/L and the electropolishing duration of 200 s. The *R*_a_ was 26.2 nm (as shown in Fig. 6b), which was much rougher than the *R*_a_ of 9.6 nm; it was obtained when the electrolyte concentration of 0.5 mol/L was used for the same duration of 200 s. The current density decreased with the electrolyte concentration owing to the reduced electrical conductivity, resulting in a lower material removal rate. Figure 12 shows the current density transitions with the NaOH concentrations of 0.27 mol/L and 0.5 mol/L, which shows that the current density with the concentration of 0.27 mol/L is half of that with the concentration of 0.5 mol/L. Therefore, the tungsten surface might not be surface treated sufficiently with the concentration of 0.27 mol/L and duration of 200 s. Figure 13 shows the electropolishing results with increased durations using the electrolyte concentration of 0.27 mol/L. As shown, the tungsten surfaces were similar for the durations of 400 s and 500 s; this implied that the tungsten surface was sufficiently electropolished after 400 s with the electrolyte concentration of 0.27 mol/L. Figure 14 shows the three-dimensional (3D) plot of the electropolished tungsten surface based on a duration of 400 s and the NaOH concentration of 0.27 mol/L. A low *R*_a_ of 7.9 nm was obtained. Figure 15 shows the *R*_a_ obtained using different NaOH concentrations. The minimum *R*_a_ decreased from 9.6 nm to 7.5 nm when the NaOH concentration was decreased from 0.5 mol/L to 0.27 mol/L. With the concentrated acid electrolyte, the effect of electrolyte concentration on the electropolishing effect is likely attributed to the concentration of the limitation species, which are responsible for the mass transportation limitation in the viscous film layer [24, 26]. However, a thick viscous film layer cannot be formed on the tungsten surface with the NaOH aqueous solution because the dissolved ions can diffuse away from the tungsten surface in time with a low viscosity of the NaOH electrolyte. Therefore, the effect of NaOH concentration on electropolishing cannot be explained by the mass transport limitation theory.

It is assumed that localization effects, which localize the electrochemical dissolution process in a small gap width, are greater when the electrolyte concentration is lower [15], enabling a more prominent electropolishing effect to be realized with a lower NaOH concentration. The anodic dissolution process favors the nearest position between the anode and cathode electrodes owing to the higher current density, and a lower electrolyte concentration reduces the effective interelectrode gap width for anodic dissolution, resulting in better localization effects and machining accuracy. Furthermore, the lower electrolyte concentration generated comparatively less sludge in the narrow working gap by not only providing lower amounts of precipitates, but also by minimizing the machining allowance. Meanwhile, Krauss et al. [38] investigated the electrochemical behavior of tungsten in electrolytes with different pH values. It was discovered that a pH value higher than 12 was not required for the electrochemical dissolution of tungsten, and reactions other than tungsten dissolution did not occur in the high pH range (approximately 10). In this study, the pH values of the NaOH aqueous solution were higher than 13 with the concentrations of 0.25 mol/L and 0.5 mol/L. Therefore, it was assumed that the slight decrease in the minimum *R*_a_ with decreasing electrolyte concentration, as shown in Fig. 15, could be limited by the high pH values of the electrolyte.

In the electropolishing of tungsten with different types of electrolytes, tungsten could not be polished in the concentrated acid electrolyte owing to the oxide film on the surface, and a clear electropolishing effect was obtained in the NaOH electrolyte. The oxide film on the tungsten surface can be dissolved by reaction (7), resulting in material removal in the NaOH electrolyte. As for the electropolishing of tungsten in the NaOH electrolyte, the current density increased with decreasing interelectrode gap width because of the decreased gap resistance. However, the current density decreased when the interelectrode gap width was extremely narrow because the generated bubbles could not escape from the working gap in time, resulting in increased gap resistance. These electropolishing characterizations exhibit similar features with those obtained from the electropolishing process with the conventional concentrated acid electrolyte. The electropolishing effect was investigated based on different interelectrode gap widths of 3, 5 and 7 mm when electropolishing 316L stainless steel with an electrolyte composed of phosphoric acid, sulfuric acid, glycerin, and deionized water [37]. The optimal electrode gap width was 5 mm; however, a smooth surface was achievable with a gap width of 7 mm. The surface roughness was worse when the gap width was 3 mm because a small gap obstructed the bubbles from escaping from the working gap. However, it was observed that the interelectrode gap width was much larger when the concentrated acid electrolyte rather than the NaOH electrolyte was used. Because of the high viscosity and a thick viscous film layer formed on the workpiece surface, a larger interelectrode gap width was required for the bubbles to escape from the working gap with the concentrated acid electrolyte.

### Preparation of copper tool electrode for nanoscale etching

**Fig. 16:**
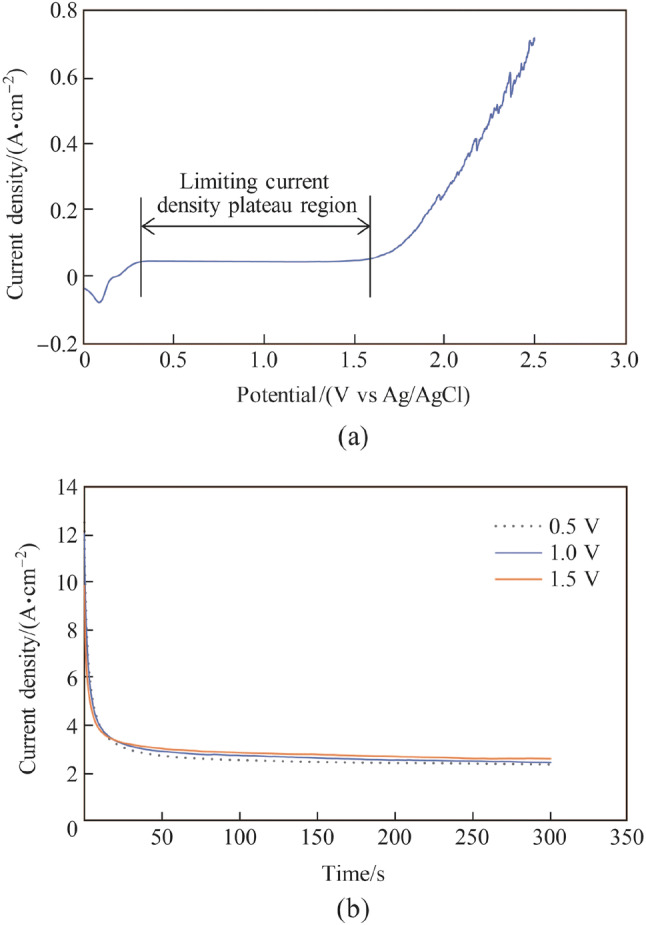
**a** Polarization curve measured with the scan rate of − 20 mV/s and potential range of 0–2.5 V and **b** current density transient with different applied potentials and electropolishing duration of 300 s

**Fig. 17:**
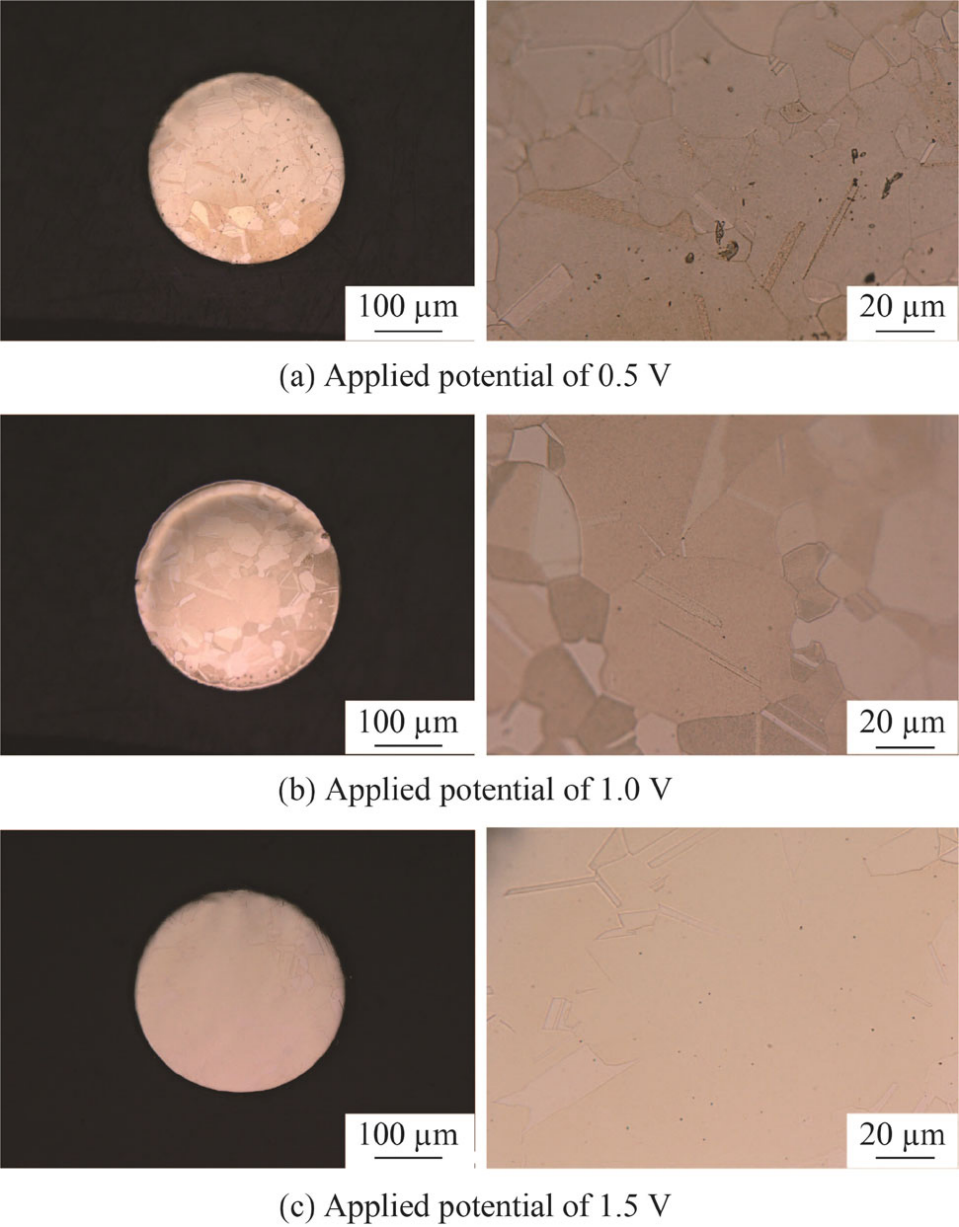
Electropolished copper surfaces with different applied potentials

**Fig. 18:**
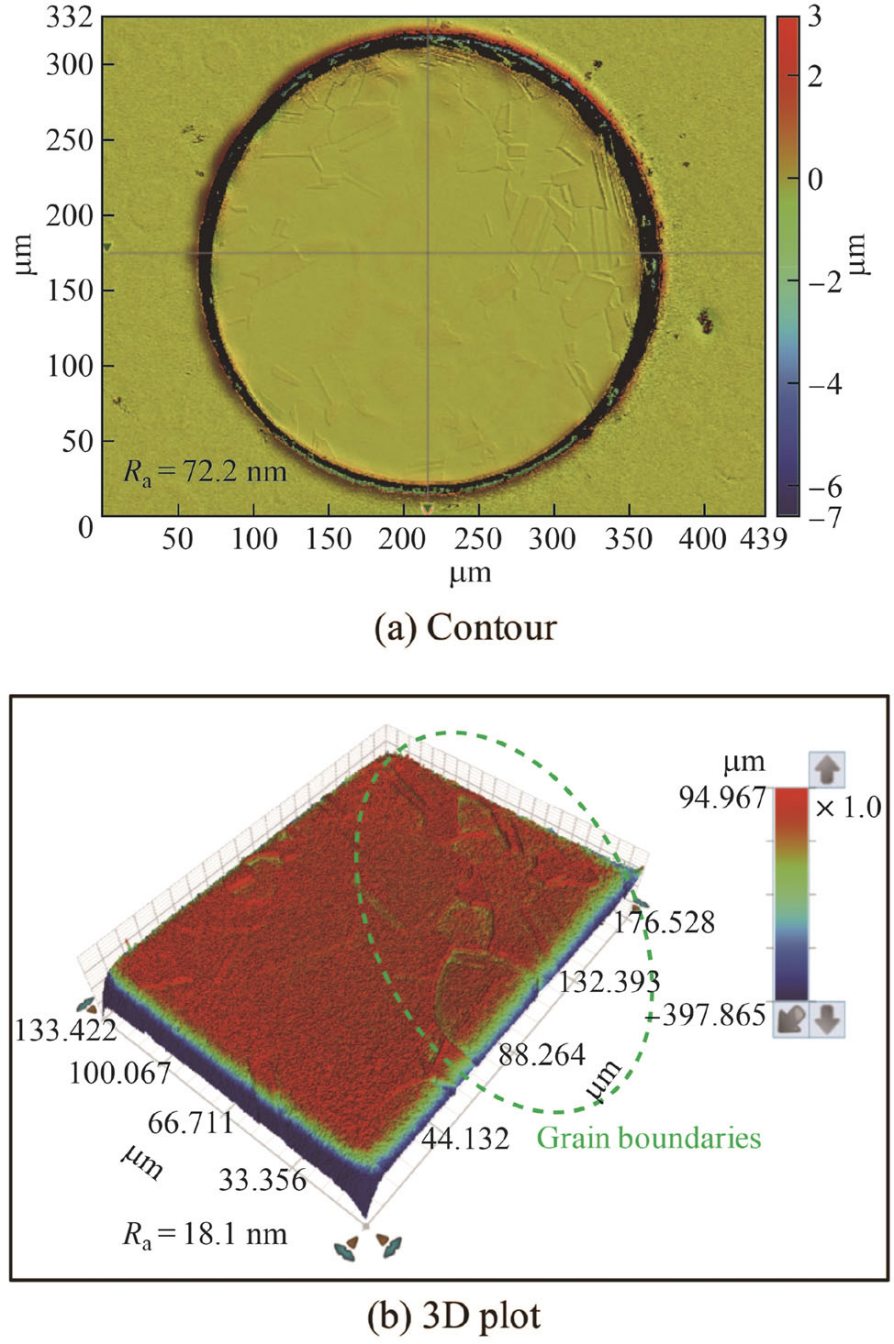
Surface counter and 3D plot of the electropolished copper surface with the applied potential of 1.5 V

Figure 16a shows the polarization curve of the copper tool electrode in the H_3_PO_4_ electrolyte. It was obtained by sweeping the applied potential from 2.5 V to 0 V (vs Ag/AgCl_sat_) at a scan rate of − 20 mV/s. A limiting current density plateau region is shown clearly in the potential range from 0.35 V to 1.6 V, and the best electropolishing effect can be obtained in this region [24]. Figure 16b shows the current density transients measured with different applied potentials along the limiting current density plateau region. The current density shows a clear passivation phenomenon at the beginning of the electropolishing, in which the current density decreases quickly with the potential. Subsequently, it shifts to a constant value with increasing electropolishing duration, which is a typical current density transient in the electropolishing process [39]. Because the mass transportation process is limited in the limiting current density plateau region, the current densities are almost the same for the magnitudes of stable current density with different applied potentials. Figure 17 shows the electropolished copper surfaces with different applied potentials corresponding to the current density transients shown in Fig. 16b. The best surface finish was obtained with the high applied potential of 1.5 V, and only a few grain boundaries remained on the electropolished surface. Figure 18 shows the surface counter and 3D plot of the electropolished copper surface with the applied potential of 1.5 V. The *R*_a_ was reduced to 18.1 nm although a few grain boundaries remained. Because the left grain boundaries did not indicate any effect in the subsequent experiments when it was directly used as a tool electrode for nanoscale etching, this electropolished copper tool was used in this study.

### Nanoscale etching of electropolished tungsten

**Fig. 19:**
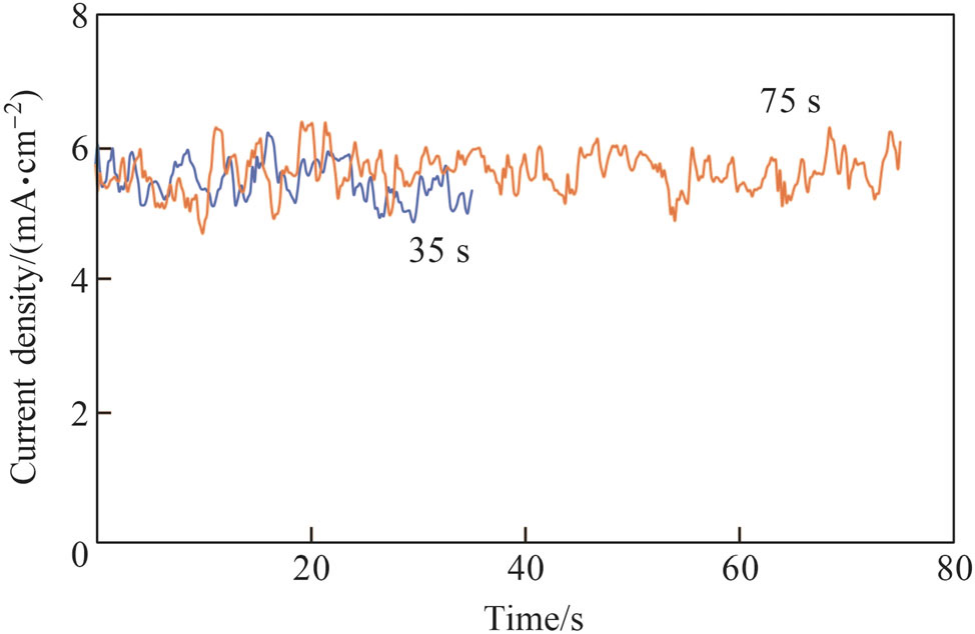
Current density transients with different etching durations

**Fig. 20:**
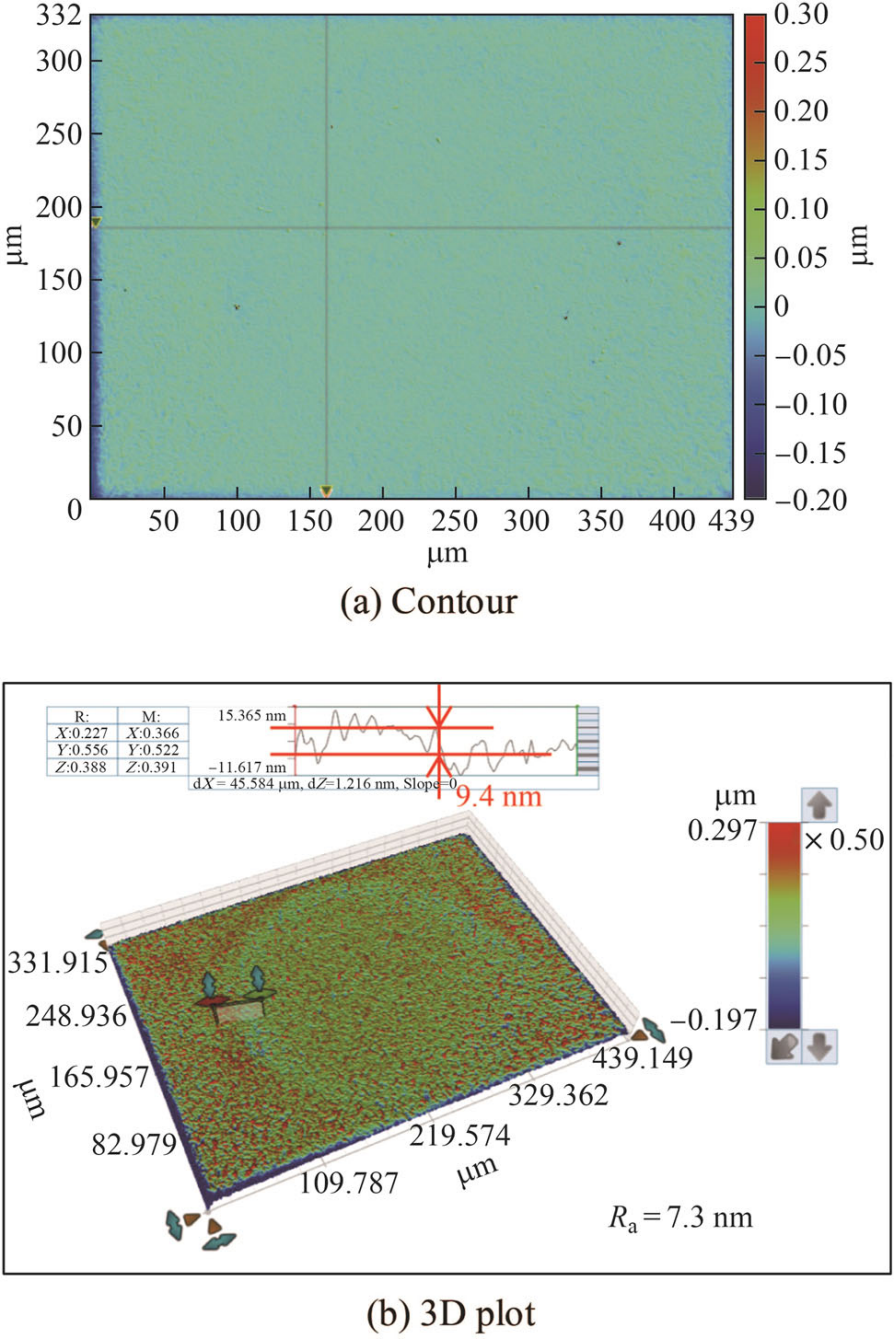
Etching results with a duration of 75 s

**Fig. 21:**
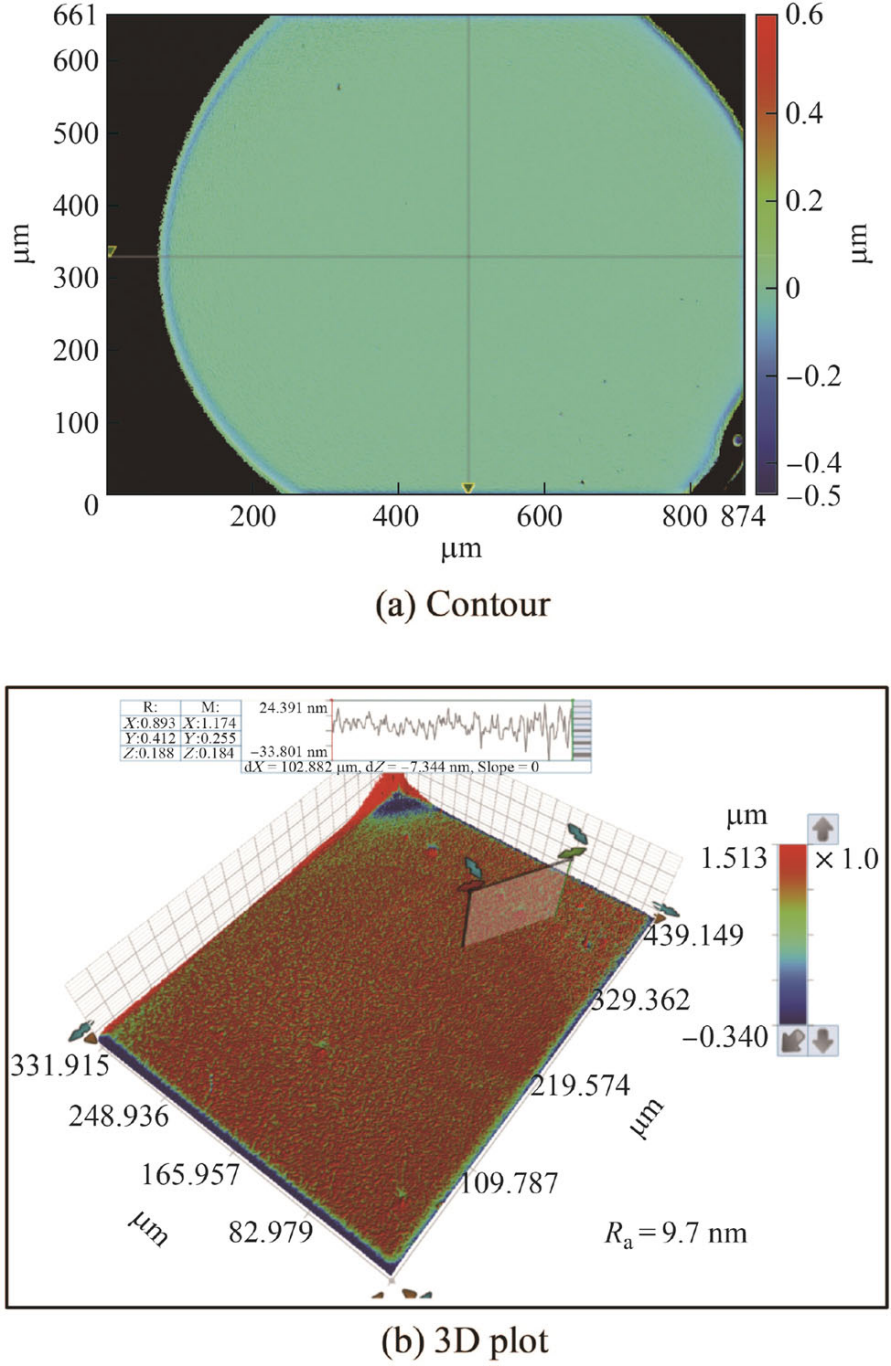
Etching results with a duration of 35 s

Figure 19 shows the current density transients with different etching durations. The current density oscillated significantly because of the effect of gas bubbles generated in the narrow working gap on the gap resistance. Figure 20 shows the results of etching for 75 s. An etched circular area was observed, and the material removal depth was 9.4 nm. Figure 21 shows the results of etching for 35 s. It was difficult to identify the etching area from the contour image; consequently, the material removal depth could not be measured effectively based on the profile, as shown in Fig. 21b.

**Fig. 22:**
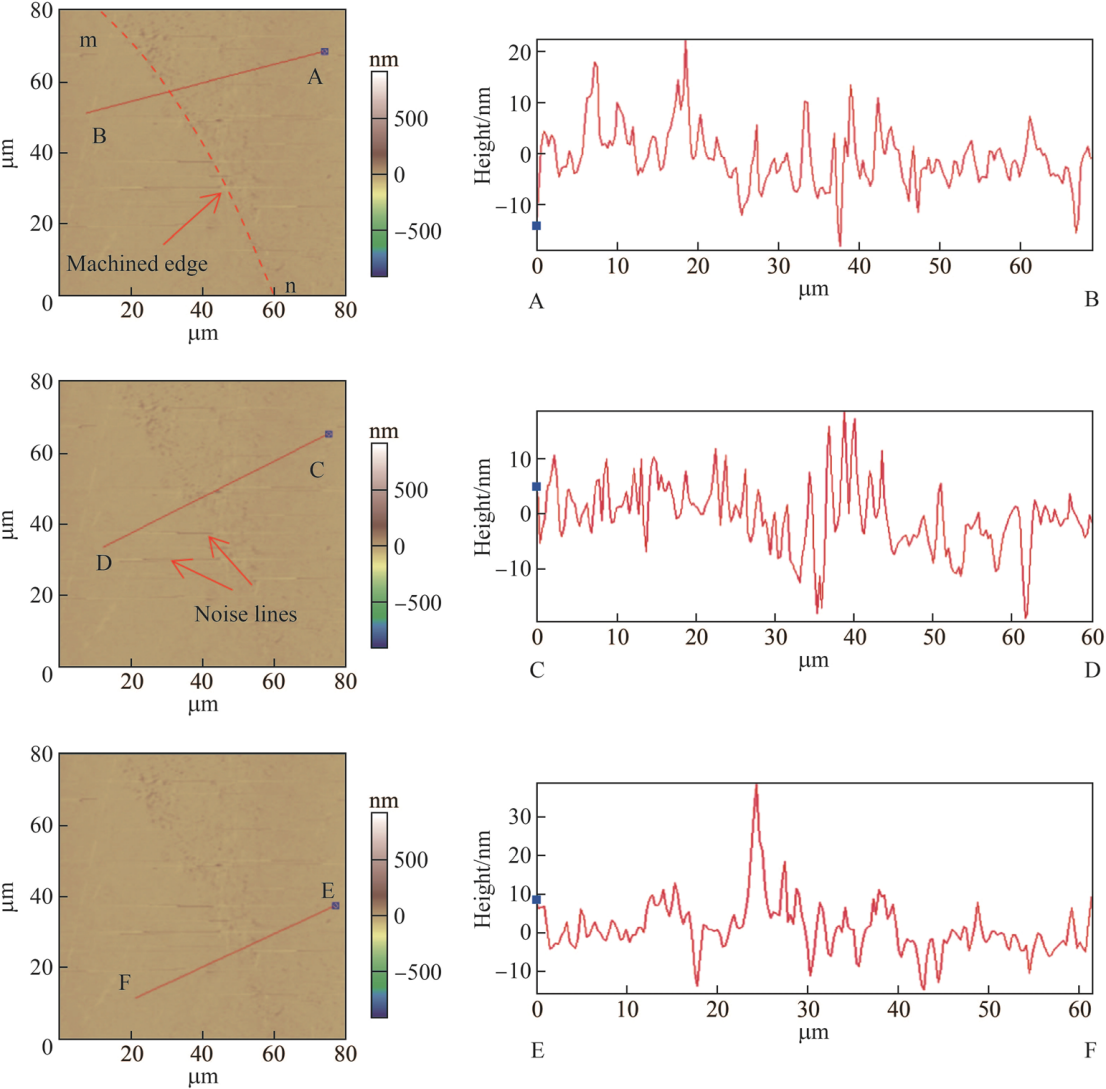
Etching results with a duration of 35 s measured by AFM (“mn” is the machined edge, “AB,” “CD,” and “EF” are the three lines crossing the machined edge “mn” at different positions)

**Fig. 23:**
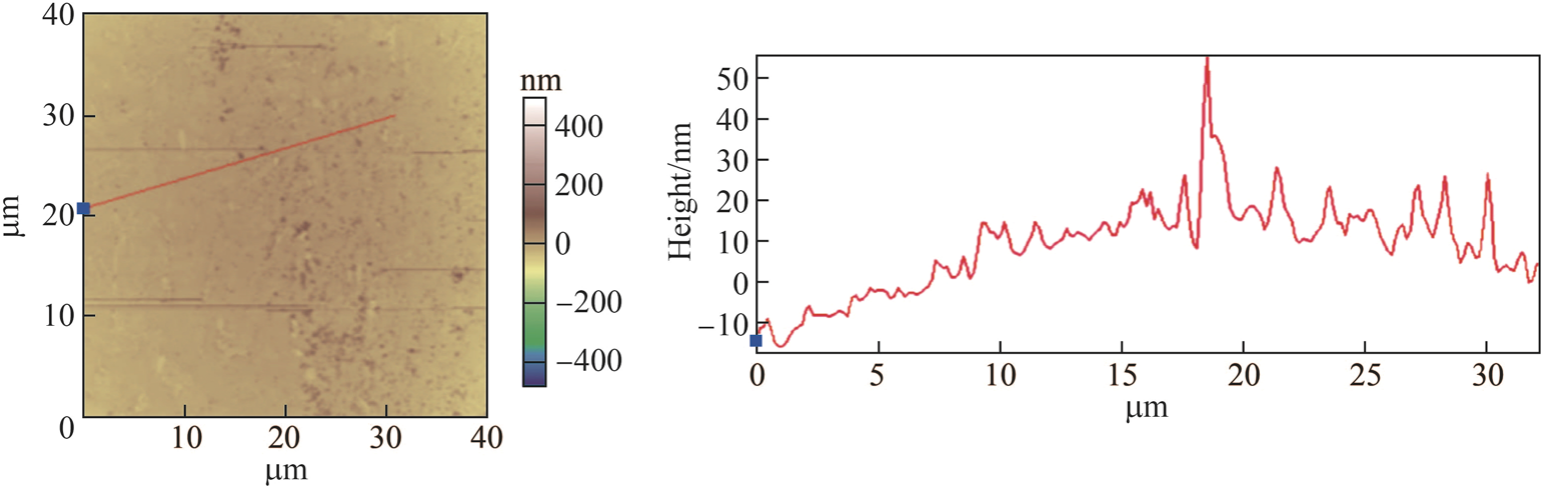
Amplified image at the etching edge with a duration of 35 s

Therefore, the tungsten etched for 35 s was measured using atomic force microscope (AFM); the results are shown in Fig. 22. Some black lines appeared owing to the effect of noise. The arc “mn” was the etched edge and the lines “AB,” “CD,” and “EF” crossed the machined edge at different positions. A material removal depth of less than 10 nm was achieved. Nonetheless, the surface finish of the electropolished tungsten should be further improved in the future because, as shown in Fig. 22, the etched edge was not clear in the three abovementioned lines because of the rough surface. Therefore, the *R*_a_ of 7.5 nm used in this study might have affected the results when the material removal depth was further decreased. Moreover, Fig. 23 shows the amplified image at the machined edge, which shows that the machined edge is not a steep step. This occurred because the etching did not have sufficient localization ability to limit the electrochemical dissolution to a sufficiently small area. It is assumed that a pulse or ultrashort pulse voltage etching can yield a smaller etching removal depth because an electrical double layer on the electrode surface can be utilized to localize the electrochemical dissolution in a small working gap [40].

## Conclusions

The electropolishing of tungsten was studied using different types of electrolytes, and the minimum material removal depth on the tungsten surface was investigated using the electrochemical etching method. A smooth surface could not be obtained when the conventional concentrated acid electrolyte was used; on the contrary, a sufficient electropolishing effect was achieved when a NaOH electrolyte was used. The following conclusions were obtained.

(i) The electropolished tungsten in a concentrated acid electrolyte indicated a slight electropolishing effect owing to the thick oxide film on the tungsten surface. Furthermore, the surface color of tungsten changed after electropolishing because of a change in the oxide film thickness.
(ii) When electropolishing tungsten in a NaOH aqueous solution, an optimum interelectrode gap width to obtain the best electropolishing effect existed. At an extremely small gap width, the current density decreased because the gas bubbles could not escape from the working gap in time, resulting in a high gap resistance. At a large gap width, the surface finish was deteriorated by the low current density owing to the large gap resistance.
(iii) The *R*_a_ decreased from 9.6 nm to 7.5 nm when the NaOH concentration was increased from 0.5 mol/L to 0.27 mol/L. Localization effects were more prominent at a lower electrolyte concentration, enabling a more pronounced electropolishing effect to be realized than when a higher electrolyte concentration was used. Furthermore, the lower electrolyte concentrations generated comparatively less sludge in the narrow working gap.
(iv) A material removal depth of less than 10 nm was achieved when the etching area measured 300 µm in diameter on the electropolished tungsten surface. In addition, the machined edge was not a steep step because the ECM did not possess sufficient localization ability to limit the material dissolution process to a sufficiently small area.
